# Implementation and outcomes of Hugo^TM^ RAS System in robotic-assisted radical prostatectomy

**DOI:** 10.1590/S1677-5538.IBJU.2023.9902

**Published:** 2022-12-20

**Authors:** Claudia González Alfano, Marcio Covas Moschovas, Vianette Montagne, Irela Soto, James Porter, Vipul Patel, Ruben Ureña, Elias Bodden

**Affiliations:** 1 Hospital Pacífica Salud Punta Pacífica Panama Hospital Pacífica Salud, Punta Pacífica, Panama;; 2 AdventHealth Global Robotics Institute Florida USA AdventHealth Global Robotics Institute, Florida, USA;; 3 University of Central Florida Florida USA University of Central Florida (UCF), Florida, USA;; 4 Swedish Medical Center Seattle Washington USA Swedish Medical Center Seattle, Washington, USA

**Keywords:** Robotics, Robotic Surgical Procedures, Prostatectomy

## Abstract

**Background:**

The results and benefits of Robotic-assisted Radical Prostatectomy (RARP) are already established in the literature. However, new robotic platforms have been released recently in the market and their outcomes are still unknown. In this scenario, our objective is to describe our experience implementing the Hugo^TM^ RAS robot and report the clinical data of patients who underwent Robotic-assisted Radical Prostatectomy.

**Material and Methods:**

We retrospectively analyzed fifteen consecutive patients who underwent RARP with Hugo^TM^ RAS System (Medtronic, Minneapolis, USA) from June to October 2021. The patients underwent transperitoneal RARP on lithotomy position, using six trocars (4 robotic trocars and 2 for the assistant). We reported the clinical feasibility and safety of this platform, assessing perioperative data, including complications and early outcomes. Continuous variables were reported as median and interquartile ranges, categorical variables as frequencies and proportions.

**Results and Limitations:**

All procedures were safe and feasible with no major complications or conversion. Median operative time was 235 minutes (213-271), and median estimated blood loss was 300ml (100-310). Positive surgical margins were reported in 5 patients (33%). The median hospitalization time was 2 days (2-2), and the median time to remove the foley was 7 days (7-7). On the first appointment four weeks after surgery, all patients had undetectable PSA values, and 61% were continent.

**Conclusions:**

We described preliminary results with safe and feasible procedures performed with Hugo^TM^ RAS System robotic platform. The surgeries were successfully executed with acceptable perioperative outcomes, without conversions or major complications. However, as this technology is very recent, further studies with a long-term follow-up are awaited to access postoperative functional and oncological outcomes.

## INTRODUCTION

The outcomes and benefits of Robotic-assisted Radical Prostatectomy (RARP) are already described and established in the literature. Since the first platform was approved by the Food and Drug Administration (FDA) in 2000, numerous models of da Vinci robots were produced in the market, and several groups described their experience with robotic surgery ( 1 - 5 ). However, only after Intuitive’s (Intuitive Surgical, Sunnyvale, CA) patent ended in 2019 different brands and models of robotic platforms were released worldwide. In this scenario, RARP with Hugo^TM^ RAS System (Medtronic, Minneapolis, USA) was approved in 2021 by the Panama healthcare regulatory agency (Ministry of Health, Minsa) for clinical use in urologic procedures.

This multiport platform has some modifications compared to the conventional da Vinci (Intuitive Surgical, Sunnyvale, CA) consoles. The arms are placed in separate karts for independent docking, while the console provides an open design with a 3D screen visualized by the 3D glasses used by the surgeon. However, due to the recent release of Hugo^TM^ RAS in the market, the literature still lacks studies describing the performance of this robot in clinical settings. In this scenario, our study describes our experience implementing the Hugo^TM^ RAS robot and the clinical data of patients who underwent Robotic-assisted Radical Prostatectomy.

## MATERIAL AND METHODS

The data of fifteen consecutive patients who underwent RARP with Hugo^TM^ RAS System (Medtronic, Minneapolis, USA) from June to October 2021 were analyzed retrospectively. All surgeries were performed by two surgeons (E.B. and R.U.) and a proctor (J.P.) using the same surgical technique and OR staff in the Hospital Pacifica Salud (Punta Pacifica, Panama). All surgeries were approved by the Hospital Internal Boards. During the preoperative consultation, the patients were advised and explained about the settings and details of this new platform, as well as the use of the data collected for analysis and studies. All patients signed a consent term of knowledge and agreement before the surgical procedure.

Respecting the patient’s privacy, the data of this study was collected with no personal identification by investigators from the center where the patients were operated (Hospital Pacifica Salud, Panama) and analyzed by investigators from AdventHealth Global Robotics Institute, USA.

We defined surgical conversion as a change in the surgical approach to laparoscopy, robotic (da Vinci), or open surgery. We reported complications according to the Clavien-Dindo classification ( 6 ). Major complications were considered as Clavien grade ≥ 3. Continence was defined as the capacity to hold urine without pads or patients using one security pad. PSA values ≥ 0.2 in two or more consecutive exams were defined as biochemical recurrence (BCR).

### Endpoints

The primary endpoint of our study is to describe the clinical feasibility and safety of the Hugo^TM^ RAS System (Medtronic, Minneapolis, USA) platform in patients who underwent robotic-assisted radical prostatectomy. We also provided a video compilation illustrating the key points of the surgery. Feasibility and safety were considered as procedures performed without conversions or major complications (see video).

The secondary endpoints were the intraoperative performance (assessed with operative time and blood loss), and perioperative outcomes from the first incision until the first postoperative visit after the catheter removal (four weeks after surgery). We also described early continence and PSA value reported in this first visit. Potency outcomes were not collected due to the short-term follow-up.

### HugoTM RAS training and robotic surgery experience

Before performing the first case with this new robot, our whole team underwent hours of training to approach the new settings and details of this technology. Each surgeon realized 17 exercises (3 times each) in a Dry Lab followed by 16 hours of system knowledge, docking, and troubleshooting. Then, the surgeons spent 16 to 20 hours performing renal and prostate surgery in cadavers while the staff members learned how to deal with the robotic arms and instruments during the procedure.

The day before the first surgery, we simulated a room set up by positioning the operative table, robotic components, and anesthesia equipment, which allowed us to save time during the clinical cases.

The surgeons involved in this study (E.B. and R.U.) are references in robotic surgery in Panama and had done more than one hundred robotic-assisted radical prostatectomies with the da Vinci console before starting Hugo^TM^ RAS training.

### Inclusion criteria

While establishing the clinical application of the Hugo^TM^ RAS robot, we selected favorable cases for RARP. We included patients with low BMI (≤ 30 Kg/m2), small prostates (≤ 70gr), no previous abdominal surgeries, and no previous prostate interventions to treat BPH or cancer. We also selected confined tumors and avoided clinical stages (cTNM) T3 or T4.

### Hugo Platform details

#### Robotic arms (individual karts)

**Figure 1:**
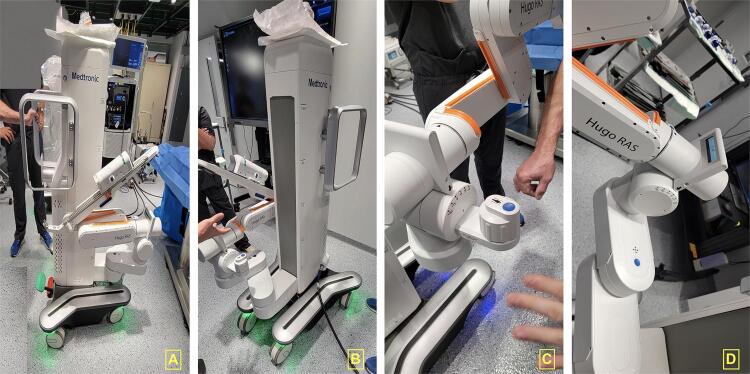
: A and B: HugoTM RAS System individual karts. C and D describing the lateral with of the karts with the angulation adjustment.

One of the modifications of this platform regards the robotic arms. Instead of all arms attached to a central tower, as the standard multiport robots, the Hugo^TM^ RAS robot has 4 independent arms attached to individual karts ( Figure-1A and Figure-1B ).

Each arm has a different docking angle ( Figure-1C and Figure-1D ) to achieve an optimal trocar placement and instrument movement during the surgery:

1. Scope (185-degree angle), Tilt - 45-degree angle
2. Right arm (230-degree angle), Tilt - 30-degree angle
3. Left arm (140-degree angle), Tilt - 30-degree angle
4. Fourth arm on the left side (105-degree angle), Tilt + 15-degree angle

## Patient positioning and trocar placement

**Figure 2:**
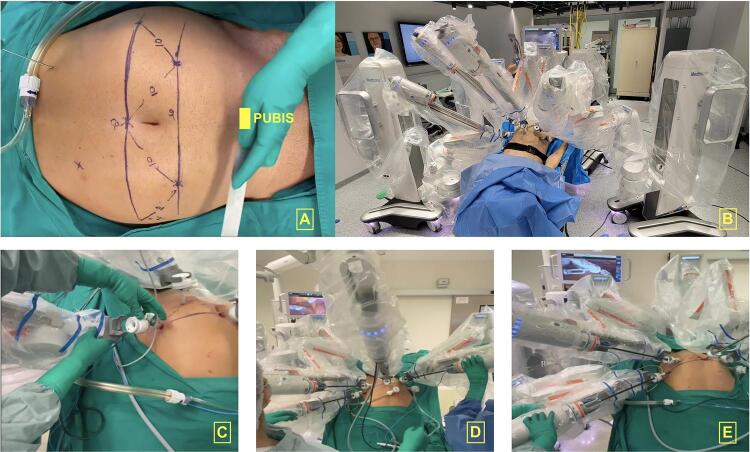
: A: port placement configuration. B: final aspect after docking. C: docking the left arm. D, and E: final aspect after instrument placement.

The patient is positioned in lithotomy to allow the placement of the scope kart between the legs. Before placing the trocars, we mark the abdomen according to Figure-2A . We initially mark 2 lines; the first is supraumbilical, 20cm from the pubis, and the second is 6 to 8 cm below the first line, on an infraumbilical position. Then, the 8mm Hugo^TM^ RAS trocars are placed respecting the 9 to 10 cm distance between the ports. After placing the scope trocar on the supraumbilical midline position (1st line), the other trocars are positioned under direct view. Finally, a 12mm assistant trocar is placed on the right lower quadrant and a 5mm trocar between the scope and the right arm.

### Docking and Instruments

**Figure 3:**
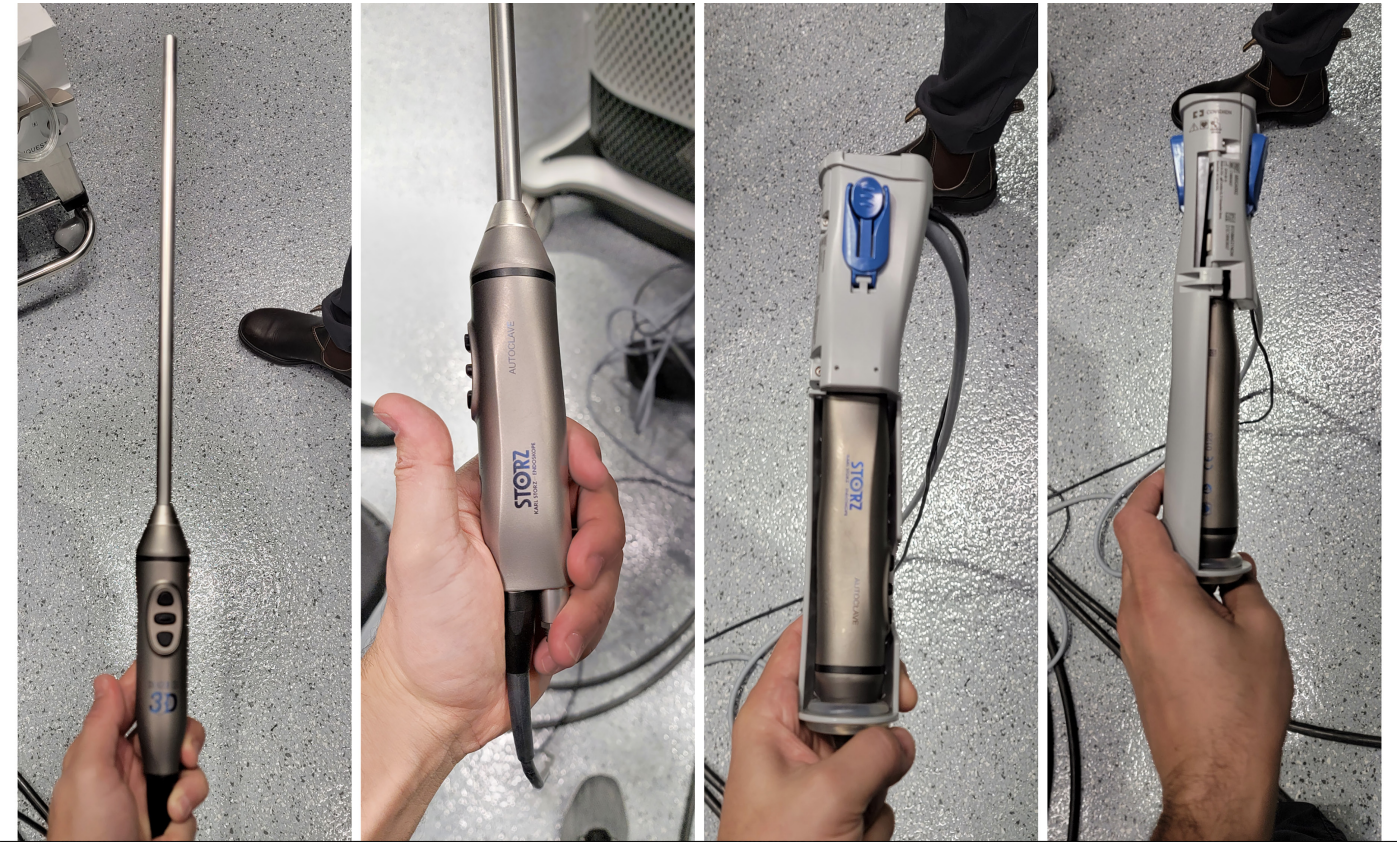
: 3D scope attached to the robotic adapter.

The docking is performed after parking each kart on the correct position and setting the appropriate angle of each arm ( Figures 2B-E ). After attaching the trocars to each arm, we place the scope, which is a 3D laparoscopic scope attached to a robotic adapter ( Figure-3 ) to fit and work with the robotic command.

### Console

**Figure 4:**
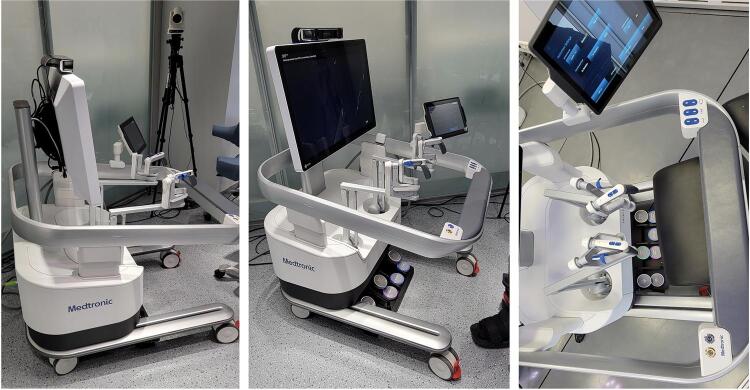
: HugoTM RAS System open console.

The console is also another innovation compared to the previous robotic platforms in the market. This robot provides an open console with a 3-dimensional view glasses for the surgeon and other visitors in the room ( Figure-4 ). The surgeon’s glasses are different than the visitors due to a security device is implanted to activate or lock the robot during surgery.

**Figure 5:**
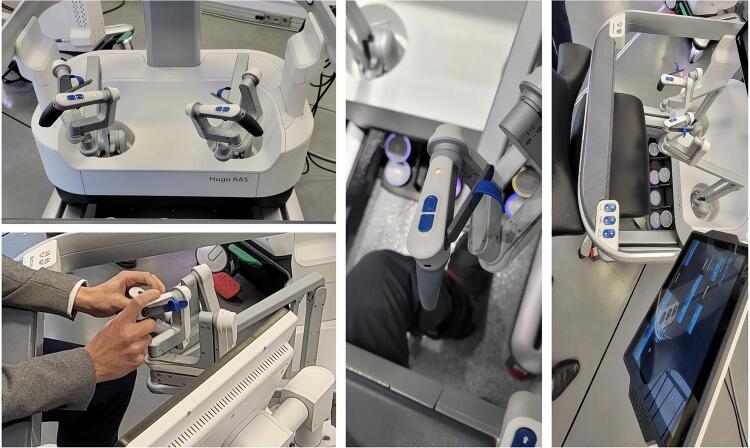
: HugoTM RAS System hand control (pistol-like).

Another modification is seen on the design and settings provided by the hand controllers, which consist of a pistol shape device with clutch on the second finger and unlocking command activated by the third finger ( Figure-5 ).

## SURGICAL TECHNIQUE

All patients underwent surgery in lithotomy position with all articulations and parts in contact with the table protected by pads. We performed a transperitoneal technique according to the previously described following steps: ( 2 , 7 - 11 ).

1. Patient positioning and trocar placement
2. Bladder dropping and Retzius space access
3. DVC control and suspension stitch
4. Anterior bladder neck dissection
5. Posterior bladder neck dissection and seminal vesicles approach
6. Nerve sparing (posterior access and lateral dissection)
7. Prostatic pedicles control with Hem-o-lock clips
8. Apical dissection and urethra division
9. Lymphadenectomy
10. Posterior reconstruction and anastomosis

### Postoperative care and follow-up

After surgery and anesthesia recovery, patients were stimulated to walk. Liquid diet was given in the afternoon of the surgery for those operated on in the morning and the next morning for those operated on in the afternoon. Compressive socks were used until ambulation in the first postoperative day. Prophylactic enoxaparin was also used from the first until the fifth postoperative day. Patients were released home in the second day after surgery (morning) and returned for Foley removal on the seventh day.

## Statistical analysis

The statistical analysis performed was based on established guidelines describing continuous variables as the median and interquartile range (IQR) ( 12 , 13 ). Absolute and percentage relative frequencies were used for categorical variables.

## RESULTS

### Preoperative demography

Table-1 illustrates the preoperative demography of this cohort. We reported median values with interquartile range (IQR) and the number of patients with the percentage. We reported the biopsy according to the International Society of Urologic Pathology (ISUP) Grade Groups (GrGp) ( 14 ).

**Table 1:** Preoperative demography of 15 patients reporting the median value with the interquartile range (IQR) and the number of patients with the percentage. PSA (Prostate Specific Antigen), BMI (Body Mass Index), ISUP (International Society of Urological Pathology).

Parameters of 15 patients	
Age (years)	62 (59 - 67)
PSA (ng/mL)	7.3 (4.8 - 8.1)
BMI (Kg/m^2^)	24.9 (23 - 28)
**Clinical Stage, n (%)**	
cT1	8 (53)
cT2a	4 (26)
cT2b	2 (13)
cT2c	1 (7)
≥cT3	0
**Biopsy ISUP grade, n (%)**	
Group 1	7 (47)
Group 2	6 (40)
Group 3	0
Group 4	2 (13)
Group 5	0

### Perioperative

Table-2 describes the perioperative outcomes. All procedures were safe and feasible with no major complications or conversion. We had only one postoperative complication (gastrointestinal bleed due to gastritis). The median operative time was 235 minutes (213-271), and the median estimated blood loss was 300ml (100-310). Positive surgical margins were reported in 5 patients (33%). The median hospitalization time was 2 days (2-2) and the median time to remove the foley was 7 days (7-7). On the first appointment four weeks after surgery, all patients had indetectable PSA values and 61% were continent.

**Table 2:** Perioperative characteristics of 15 patients reporting the median with the interquartile range (IQR) for continuous variables and the number of patients with percentage for categorical variables. ISUP (International Society of Urological Pathology).

Parameters in 15 patients	
EBL (mL)	300 (100-310)
Total operative time (minutes)	235 (213 - 271)
Lymphadenectomy n, (%)	5 (33)
Intraoperative Complications n, (%)	0
Postoperative Complications n, (%) *	1(6)
Positive Surgical Margins n, (%)	5 (33)
Pathological Stage n, (%)	
pT2	11 (74)
pT3	4 (26)
**Final Pathology ISUP grade, n (%)**	
Group 1	2
Group 2	11
Group 3	1
Group 4	0
Group 5	1
Prostate volume (cc)	52 (41-56)
Hospital Stay (days)	2 (2-2)
Time to remove Foley (days)	7 (7-7)
**Continence in 4 weeks n, (%)**	
Continent	9 (61)
Stress incontinence	5 (33)
Not continent	1 (6)
Undetectable PSA in 4 weeks n, (%)	15 (100)
Follow-up (weeks)	4 (4-4)

## DISCUSSION

In the recent years, after the end of Intuitive’s (Intuitive Surgical, Sunnyvale, CA) exclusivity in the robotic surgery field, several brands, and models of multiport and single-port robots were released in the market with promising technology ( 15 - 20 ). However, as most of them are still under a validation process, the literature still lacks robust data describing the performance and outcomes of these new platforms in urologic procedures. In this scenario, our study described the first clinical experience and perioperative outcomes of 15 patients who underwent robotic-assisted radical prostatectomy with Hugo^TM^ RAS System (Medtronic, Minneapolis, USA).

Using new technologies to operate patients in clinical settings is always challenging ( 17 , 19 ). However, before the implementation of this robot in our center, our team had previous expertise with robotic surgery after performing numerous cases of radical prostatectomy with the da Vinci console (Intuitive Surgical, Sunnyvale, CA). In addition, Panama was one of the first countries in the world to approve this robot for clinical use and our hospital (Hospital Pacifica Salud) was the first to acquire this technology to approach General Surgery, Gynecologic, and Urologic surgeries. In our experience, the main challenge during the implementation process was the learning curve of staff and surgical team associated with the modified docking and some console settings.

The patient positioning (lithotomy) and trocar placement are very similar to the da Vinci platform (Intuitive Surgical, Sunnyvale, CA). The appropriate distance and angles between the trocars must be respected to achieve the correct triangulation and instrument movement. However, the docking process is more challenging and demands training because all arms are attached to individual karts that must be placed in the correct position with an appropriate arm angulation. If these parameters are not respected, the optimal angles and arm movements will be compromised during the surgery. The first docking had the longest time (approximately 15 minutes) due to the setup of the karts. Then, we had a median time of 7 minutes docking per case in the following procedures.

In our first impression, the open console and new design of the hand controls could be faced as a challenge to our learning curve due to years of experience in a different platform with another operative setting. However, once the robot is docked and the instruments are placed, the high-definition 3D image provided by the 3D glasses did not change our approach to the surgery. In addition, by using extra glasses, other surgeons and visitors around the console can see the same operative 3D image as the surgeon. We also believe that the hand commands (pistol-like) and settings did not interfere in the surgical technique, but it demands an adaptive period until mastering the different buttons to lock and unlock the arms.

During consecutive steps of robotic-assisted radical prostatectomy, we believe that the instruments provided appropriate traction and dissection capacity without delaying or interfering on the intraoperative performance. The operative time is compatible with what we usually perform in other robotic platforms, and we did not have any operative complications related to the robotic technology. However, as this robot is still new in the market, and not available in most countries yet, we still need a longer follow-up to assess functional and oncological outcomes compared to other consoles.

Despite its strengths, our study is not devoid of limitations, especially due to its retrospective design and all its inherent risk of bias. In addition, the small number of patients and lack of a comparison group limits the analysis of outcomes compared to other platforms. Also, the short-term follow-up restricts the assessment of functional and oncological outcomes. However, to the best of our knowledge, this is one of the first clinical reports of Hugo^TM^ RAS System application in Robotic-assisted Radical Prostatectomy. Our study provided data describing safe and feasible procedures with acceptable short-term continence recovery, which is in line with our primary endpoints. We did not assess long-term results due to the short period of this console in the market. Finally, we believe that the illustrations and data of this study are crucial for understanding the first steps of the implementation process of this new technology.

## CONCLUSIONS

We reported the clinical application of Hugo^TM^ RAS System in patients who underwent radical prostatectomy. Our data described preliminary results with safe and feasible procedures performed with this novel robotic platform. The surgeries were successfully performed with acceptable perioperative outcomes and without conversions or major complications. However, as this technology is very recent, further studies with a long-term follow-up are awaited to access postoperative functional and oncological outcomes.
